# Embryonic Stem Cells Promoting Macrophage Survival and Function are Crucial for Teratoma Development

**DOI:** 10.3389/fimmu.2014.00275

**Published:** 2014-07-04

**Authors:** Tianxiang Chen, Xi Wang, Lei Guo, Mingmei Wu, Zhaoxia Duan, Jing Lv, Wenjiao Tai, Hemamalini Renganathan, Ruth Didier, Jinhua Li, Dongming Sun, Xiaoming Chen, Xijing He, Jianqing Fan, Wise Young, Yi Ren

**Affiliations:** ^1^W. M. Keck Center for Collaborative Neuroscience, Rutgers, The State University of New Jersey, New Jersey, NJ, USA; ^2^Department of Thoracic Surgery, First Affiliated Hospital, School of Medicine, Zhejiang University, Hangzhou, China; ^3^Institute of Neurosciences, The Fourth Military Medical University, Xian, China; ^4^Department of Biomedical Sciences, Florida State University College of Medicine, Tallahassee, FL, USA; ^5^Department of Orthopedic Surgery, The Second Hospital of Xian Jiaotong University, Xian, China; ^6^Department of Anatomy and Developmental Biology, Monash University, Clayton, VIC, Australia; ^7^Institute of Translational Medicine, First Affiliated Hospital, Wenzhou Medical University, Wenzhou, China; ^8^Statistics Laboratory, Princeton University, Princeton, NJ, USA

**Keywords:** angiogenesis, apoptosis, embryonic stem cells, macrophages, teratoma

## Abstract

Stem cell therapies have had tremendous potential application for many diseases in recent years. However, the tumorigenic properties of stem cells restrict their potential clinical application; therefore, strategies for reducing the tumorigenic potential of stem cells must be established prior to transplantation. We have demonstrated that syngeneic transplantation of embryonic stem cells (ESCs) provokes an inflammatory response that involves the rapid recruitment of bone marrow-derived macrophages (BMDMs). ESCs are able to prevent mature macrophages from macrophage colony-stimulating factor (M-CSF) withdrawal-induced apoptosis, and thus prolong macrophage lifespan significantly by blocking various apoptotic pathways in an M-CSF-independent manner. ESCs express and secrete IL-34, which may be responsible for ESC-promoted macrophage survival. This anti-apoptotic effect of ESCs involves activation of extracellular signal-regulated kinase (ERK)1/2 and PI3K/Akt pathways and thus, inhibition of ERK1/2 and PI3K/AKT activation decreases ESC-induced macrophage survival. Functionally, ESC-treated macrophages also showed a higher level of phagocytic activity. ESCs further serve to polarize BMDMs into M2-like macrophages that exhibit most tumor-associated macrophage phenotypic and functional features. ESC-educated macrophages produce high levels of arginase-1, Tie-2, and TNF-α, which participate in angiogenesis and contribute to teratoma progression. Our study suggests that induction of M2-like macrophage activation is an important mechanism for teratoma development. Strategies targeting macrophages to inhibit teratoma development would increase the safety of ESC-based therapies, inasmuch as the depletion of macrophages completely inhibits ESC-induced angiogenesis and teratoma development.

## Introduction

Stem cell-based therapies possess promising outcomes for many conditions, including spinal cord injury and other neurological degenerative disorders. However, this powerful therapeutic strategy is problematic because the pluripotency of stem cells is accompanied by a large risk of tumor formation after transplantation. Theoretically, three classes of tumors can be envisaged to arise from pluripotent stem cells (PSCs) including embryonic stem cells (ESCs) and induced pluripotent stem cells (iPSCs); viz. teratomas, teratocarcinomas, and secondary tumors (1). Teratoma is constituted by cells from endodermal, mesodermal, and ectodermal lineages (2–4). Not only ESCs but also ESC-derived neuronal progenitors can induce teratomas in animal models (5). The tumorigenicity of stem cells is the major obstacle to the successful application of stem cell-based therapies (6). The safety issue of stem cells must be evaluated properly and the adverse consequences of teratoma formation from these stem cells must be overcome before stem cell therapy can be used for clinical application.

Various strategies developed to reduce this risk of teratoma formation include prolonged pre-differentiation of ESCs *in vitro*, blocking signaling pathways that promote proliferation, induction of apoptosis of proliferative ESCs, sorting cells expressing precursor markers, and deleting undifferentiated ESCs immunologically, genetically, and chemically (7–18). However, it is difficult to obtain a yield of 100% pure differentiated stem cells for transplantation: the contamination of grafts with undifferentiated cells can give rise to teratoma formation (19–21). Furthermore, teratoma could potentially develop into highly malignant teratocarcinoma, which constitutes of persistent and undifferentiated stem cells (22). Therefore, efforts must be made to ensure safe transplantation of a PSC-based cell treatment. We used undifferentiated ESCs as a worst-case model for teratoma formation by stem cells and studied the role of macrophages and niche microenvironment of stem cell growth in the progression of teratomas.

The interplay of immune cells, especially macrophages and ESCs, causes alterations in the microenvironment and potential for tumorigenicity, which regulate the initiation, progression, angiogenesis, and metastasis of tumor. Thus, targeting this immune response can significantly inhibit the evolution of tumors (23, 24). Our previous data also demonstrated that interaction between transplanted ESCs and macrophages creates a microenvironment that facilitates the initiation and progression of teratomas (24). Infiltrated macrophages deliver macrophage migration inhibitory factor (MIF) and other angiogenic factors to stimulate endothelial cell proliferation and pericyte differentiation (24). There is growing evidence to suggest that macrophages promote tumorigenesis and that the tumor microenvironment polarizes macrophages toward an M2 (pro-tumor) phenotype, with properties that differ from the M1 phenotype (25–28). However, the role of macrophages in ESC growth and teratoma development is not clear. In this study, we demonstrate that ESCs promote macrophage survival and M2-like activation are critically important for teratoma angiogenesis and development. Significantly, we show that depletion of macrophages inhibits teratoma growth tremendously. Therefore, ESC-educated macrophages are considered attractive targets for an anti-teratoma strategy after ESC transplantation.

## Materials and Methods

### Reagents and antibodies

All the chemicals were purchased from Sigma (St. Louis, MO, USA) and cell culture media were purchased from Invitrogen (Carlsbad, CA, USA) unless specifically noted. The F4/80 hybridoma cell line was from American Tissue Culture Collection (ATCC, Manassas, VA, USA). Recombinant mouse IL-34 was from R&D Systems (Minneapolis, MN, USA). The primary antibodies used in the study are listed in Table 1. All Alexa Fluor- or HRP-conjugated secondary antibodies were from Invitrogen.

**Table 1:** **Antibodies included in the study**.

Protein name	Antibody ID	Manufacturer
AKT	4685	Cell Signaling
Arginase-1	sc-18354	Santa Cruz
Caspase-9	9504	Cell Signaling
CD31	553708	BD Biosciences
CD45	103108	BioLegend
Cytochrome *c*	4272	Cell Signaling
ERK1/2	4695	Cell Signaling
GAPDH	2118	Cell Signaling
IBA-1	019-19741	Wako
IL-34	PAB13397	Abnova
M-CSF	3155	Cell Signaling
PI3K p85	4257	Cell Signaling
Iκbα (Ser32)	2859	Cell Signaling
Phospho-Akt	9271	Cell Signaling
Phospho-ERK1/2	4370	Cell Signaling
Phospho-PI3K p85	4228	Cell Signaling
Tie-2	sc-9026	Santa Cruz
YM1	01404	Stem Cell Technologies

### Mice

C57BL/6 mice and transgenic CX3CR1^GFP^ mice from Jackson Laboratory (Bar Harbor, ME, USA) were maintained in the pathogen-free animal facility in Rutgers, the State University of New Jersey. All animal experimental protocols were authorized by the Animal Care and Facilities Committee of Rutgers, the State University of New Jersey and Florida State University.

### Preparation of mouse bone marrow-derived macrophages

Mouse bone marrow-derived macrophages (BMDMs) from C57BL/6 mice were prepared as described (24). Briefly, BM cells from mice 6–8 weeks of age were collected from femoral shafts by flushing the marrow cavity of femurs with Dulbecco’s modified eagle medium (DMEM) supplemented with 1% fetal bovine serum (FBS). The cell suspensions were passed through an 18-gage needle to disperse cell clumps. Cells were cultured for 7 days at a cell density of 1 × 10^6^/ml in 100 mm polystyrene tissue culture dishes (BD Biosciences) containing DMEM supplemented with 15% conditioned medium from L929 cells [a source of macrophage colony-stimulating factor (M-CSF)] and 10% FBS. Cell morphology was analyzed by image capture (Carl Zeiss, Germany) and using LSM 510 software (Nikon, Japan). The long axis, defined as the longest length of the cells, was manually traced and measured.

### ESC culture and preparation of ESC-conditioned medium

The mouse green fluorescent protein (GFP)-expressing -ESC line (F12) derived from C57BL/6 mouse was a kind gift from Professor Melitta Schachner (Rutgers University). Freshly thawed ESCs (P0) were seeded into a 10-cm tissue culture dish in the presence of mitomycin-treated murine embryo fibroblast (MEF) feeder layer, in ESC media [10^3^ U/ml LIF (Millipore, CA, USA), 15% FBS, 1% non-essential amino acids solution (MEM), 200 mM l-glutamine, 1% nucleoside solution, 1% 100 nM Na-Pyruvate, and 0.2% 2-Mercaptoethanol in DMEM]. LIF was added every day into the culture medium. After 3–4 passages, ESCs were maintained only on a 0.1% gelatin-coated tissue culture dish without a feeder layer. ESC colonies were sub-cultured for every 2–3 days and their supernatant was collected as conditioned medium. ESC-conditioned medium (ESC-CM) was prepared by spinning the ESCs at 1,000 rpm for 5 min to pellet the cells, while the supernatant was again spun at 2,500 rpm for 10 min to remove any debris. Supernatant was then filtered through a 0.4-μ filter (Corning, USA). Supernatant collected from multiple passages was pooled together and stored at −80°C. Regular culture medium without ESCs was incubated in the same way and used as control medium (Con-M).

### Histology and immunofluorescence

Mice were transcardially perfused with 0.9% saline followed by 4% paraformaldehyde. A segment of tissue encompassing the transplantation site was removed and fixed in 4% paraformaldehyde for 3 h and then cryoprotected in 20% sucrose overnight at 4°C. For histologic examination, the sections were stained with hematoxylin and eosin (H&E). For immunofluorescence staining, the sections were incubated with primary antibodies overnight at room temperature (RT) followed by secondary antibodies at RT for 2 h. Non-specific binding was excluded by using secondary antibody only. Samples were examined and microphotographs were taken using a Zeiss AxioCam microscope and an AxioPhot image collection system (Carl Zeiss, Germany), and Confocal Laser Scanning Microscopy (Nikon, Japan). Tumor volume was determined as follows: short diameter^2^ × long diameter × 1/2.

### Transplantation and ESCs in spinal cord and liver

Laminectomy was performed on WT and chimerical mice at the T9–T10 level to expose the spinal cord. GFP–ESCs (50,000 in 1 μl DMEM) were injected slowly at this segment of each mouse’s spinal cord using a microliter syringe (Hamilton Company, NV, USA) fixed in a stereotaxic frame. ESCs (100,000 in 5.0 μl DMEM) were also injected slowly via microliter syringe into the left lobe of mouse liver. Mice were sacrificed and perfused at different time points after cell transplantation.

### MTT assay

To determine cell viability the colorimetric MTT metabolic activity assay was used. BMDMs were seeded in a 96-well plate at a density of 8,000 cell per well and cells were treated with Con-M and ESC-M for 48 h. MTT [3-(4,5-dimethylthiazolyl-2)-2,5-diphenyltetrazolium bromide] was added to each well and plate was incubated for 4 h. Finally, the absorbance was measured at 540 nm by using a microplate reader. The relative MTT uptake (% cell viability) was expressed as a percentage relative to the control cells.

### Phagocytic function test

Mature BMDMs were seeded in a 96-well culture plate at a density of 1 × 10^4^ cells/well and incubated with Con-M and ESC-M for 24 h. BMDMs were incubated with carboxylate-modified fluorescent red Latex beads and apoptotic cells for 1 h, respectively. Non-ingested particles were washed away and phagocytosis was imaged by a Zeiss Axiovert 200 M Microscope (Carl Zeiss) and software AxioVision 4.6 (Carl Zeiss).

### Arginase activity assay

To prepare the cell lysate for assay of arginase activity, BMDMs were rinsed with PBS after each specific treatment and 1 × 10^5^ BMDMs from each group were lysed in 100 μl of lysis buffer containing 10 mM Tris-HCl (pH 7.4), proteinase inhibitor cocktail, and 0.4% Triton-X 100 for 10 min. Arginase activity of various cell lysate was measured by quantitative colorimetric assay of arginase activity (Bioassay Systems, CA, USA) according to the manufacturer’s instructions. One unit of arginase activity is defined as 1 μmol of l-arginine converted to ornithine and urea per minute at pH 9.5 and 37°C. Urea concentration, as the degree of arginase activity, was measured at 520 nm at RT by spectrometer.

### Annexin V and propidium iodide staining

Bone marrow-derived macrophages were rinsed with PBS after each specific treatment and the apoptosis of BMDMs was measured by PE Annexin V Apoptosis Detection Kit I (BD Biosciences, CA, USA) according to the manufacturer’s instructions. Binding of Annexin V and PI was measured by a flow cytometer (BD Biosciences, CA, USA) and analyzed using FlowJo software (FlowJo, NJ, USA).

### RNA isolation and quantitative real-time-PCR

Bone marrow-derived macrophages were incubated with Con-M and ESC-M for 6 and 12 h, respectively. Total RNA was isolated by TRIZOL and reverse-transcribed into cDNA by using oligo-dT primers and SuperScript II Reverse Transcriptase. The TNF-α primer pair (5′-ATGCTGGGACAGTGACCTGG-3′ and 5′-CCTTGATGGTGGTGCATGAG-3′) was specifically designed for mRNA. The ABI7900HT detection system (Applied Biosystems, UK) was used for quantitative real-time (qRT)-PCR. SYBR Green dye (Applied Biosystems) was used to monitor the replication of PCR products. Quantification of products were obtained by standard curve and then normalized to GAPDH amount. The gene expression level was represented by the ratio of gene TNF-α/GAPDH.

### Western blot

Western blot assay was performed following the standard procedure. Briefly, after washing with ice-cold PBS, cells were lysed with RIPA buffer containing phosphatase inhibitor and proteinase inhibitor cocktail. Total cellular proteins were loaded onto SDS-polyacrylamide gel electrophoresis (SDS-PAGE) and then transferred to PVDF membranes (GE Healthcare, UK). After blocking in 5% milk or BSA (according to antibody manufacturer’s instructions) in Tris-buffered saline containing 0.1% Tween 20 (TBST) for 1 h at RT, membranes were incubated with appropriate primary antibody solution overnight at 4°C. Membranes were placed in appropriate secondary antibody for 1 h after rinsing in TBST. Subsequently, proteins were visualized by ECL plus western blot detection system (GE Healthcare, UK).

### *Ex vivo* mouse aortic ring assay

Mouse aortic ring assay was carried out as described (29) using C57BL/6 mice (8–12 weeks). Briefly, thoracic aortic segments were cut into 1-mm rings and carefully placed with the lumen of the rings opened up on Matrigel (BD Biosciences) with Con-M or ESC-M and then overlaid with an additional Matrigel. Aortic rings were examined daily and digital images were taken at day 6 for quantitative analysis of the area of vessel outgrowth by the SPOT Advanced program (Media Cybernetics, Sterling Heights, MI, USA). Microvessel outgrowth was calculated by circling the extent of microvessel outgrowth at 6 days and subtracting the area of the aortic ring (29).

### Depletion of macrophages *in vivo*

Liposomes containing either dichloromethylene diphosphonate (clodronate, a gift from Roche Diagnostics, Germany) or PBS were prepared as described (30). Mice were injected with clodronate liposomes (CL-Lip) or PBS liposomes (PBS-Lip) at 100 mg/kg body weight by intraperitoneal (i.p.) injection twice a week for up to 4 weeks.

### Statistical analysis

Results showed in figures were presented as mean ± SEM with *n* representing the frequency of experiments. Student’s unpaired *t*-test was used to evaluate statistical significance with a *p* value <0.05 considered significant.

## Results

### Teratoma development after ESC injection into spinal cord

**Figure 1:**
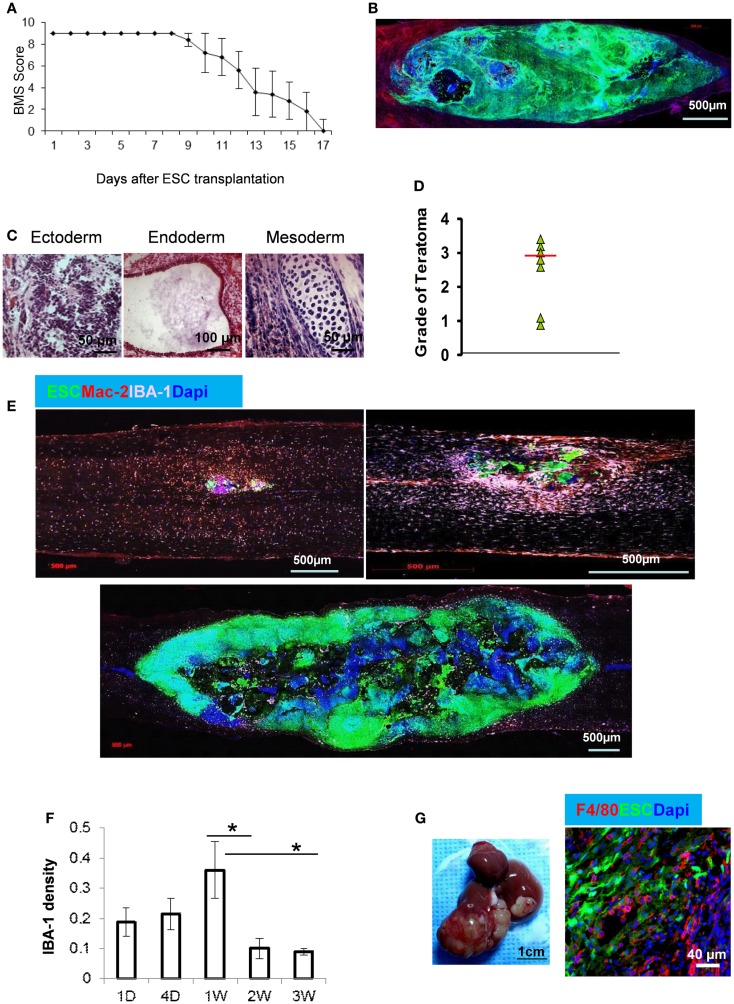
**Teratoma formation and macrophage infiltration after ESC injection into spinal cord**. **(A)** ESCs were stereotaxically injected into the spinal cord in C57BL/6 mice and the function of the hindlimbs was evaluated by BMS score (*n* = 7, data are represented as mean ± SEM). A score of 0 indicates complete paralysis of the hind limbs and 9 denotes full mobility. **(B)** Tumor formation in spinal cord at 3 weeks after GFP–ESC injection. **(C)** Histological staining of spinal cord sections at 2 weeks after ESC injection showing structures derived from three embryonic germ lineages. **(D)** Median of teratoma grade in mice (*n* = 6). **(E)** Representative micrographs showing macrophage recruitment during teratoma progression. Macrophages in the sections of spinal cord at 1 day (upper left), 1 week (upper right), and 3 weeks after ESC transplantation (lower) were detected by antibodies to IBA-1 (purple) and Mac-2 (red). **(F)** Quantification of IBA-1^+^ macrophages at indicated time points after ESC transplantation (*n* = 10, *p < 0.05, two-sided Wilcoxon test. Data are represented as mean ± SEM). **(G)** Representative gross morphology of teratoma in liver (left) and F4/80^+^ macrophages in teratoma at 4 weeks after ESC transplantation in liver.

Undifferentiated enhanced gene fluorescent protein (EGFP)–ESCs were stereotaxically injected into the spinal cord of mice exposed by a T9–T10 laminectomy. During the first week after ESC injection, hindlimb function, as reflected by the Basso Mouse Scale (BMS), was normal. However, the BMS score decreased rapidly at 10 days after ESC injection and all mice were paralyzed at day 17 after cell transplantation (Figure 1A) because of rapid tumor growth (Figure 1B). The mice survived for only 3 weeks after ESC transplantation (data not shown). Histological examination revealed that these tumors were teratomas since they consisted of structures derived from all three embryonic germ lineages (Figure 1C). While most teratomas are benign, malignant teratomas do occur. Prognosis is inversely related to stage and histological grade, which is based on the amount of neurepithelium and immature neural tubes present according to the World Health Organization (WHO) classification (31). Teratomas of grade 0–1 are classified as benign or low grade, while grade 3 is malignant. We found that the median teratoma grade in mice was 3.0 (Figure 1D), indicating that these teratomas in mice were teratocarcinomas.

### ESCs stimulate macrophage infiltration

We observed the early infiltration of a large population of Mac-2^+^/IBA-1^+^ macrophages within the teratomas (Figure 1E). Macrophages can be detected as early as 1 day after ESC injection, with peak macrophage infiltration occurring after 1 week (Figures 1E,F). Figure 1F shows the mean density of macrophages (IBA-1^+^) recruited at the ESC implantation site at different time points after ESC injection. Interestingly, there was a significant reduction in macrophage distribution after 2 weeks. The numbers of macrophages at 2 and 3 weeks were significantly less than those at any earlier time points and the difference between 2 and 3 weeks was no longer significant (Figure 1F). This suggests that macrophages may play an important role in teratoma initiation. By contrast, injection of PBS alone in the spinal cord did not induce macrophage infiltration (data not shown).

### Teratoma development in the tissue outside of spinal cord

To better exclude the effect of neural and glial cells in spinal cord on teratoma growth and differentiation, an examination of teratomas induced by ESC transplantation in non-neural sites could support the role of signals produced by macrophages vs. other tissue types. ESCs were injected into liver and representative teratoma at week 4 is shown in Figure 1G. A large teratoma had formed in the liver and an enormous number of macrophages was detected (Figure 1G).

### ESC-secreted factors act as macrophage survival factors

**Figure 2:**
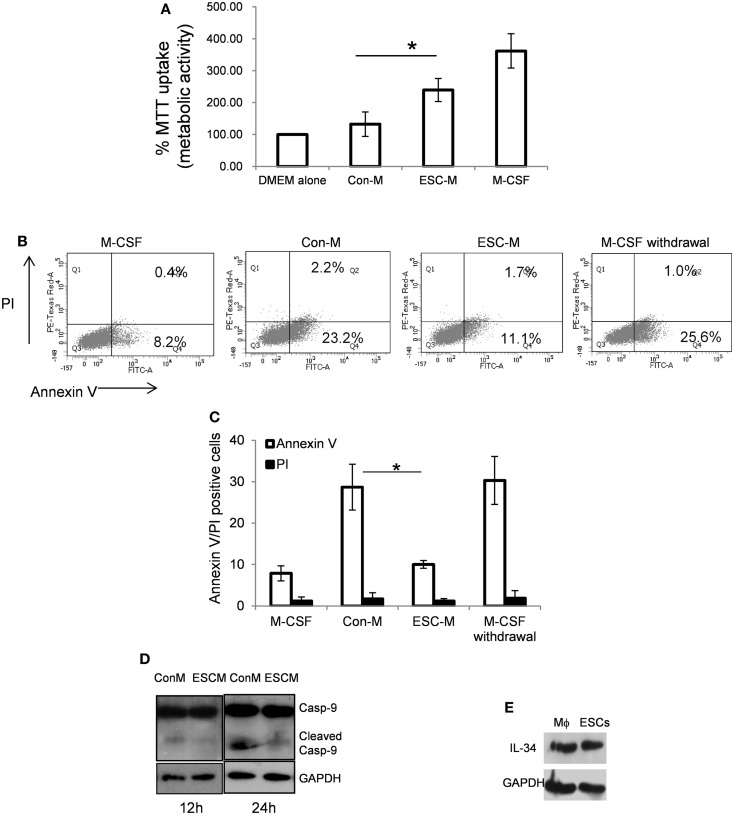
**Embryonic stem cells enhance BMDM survival**. **(A)** Day 7 BMDMs were incubated with DMEM alone in absence of M-CSF, Con-M, ESC-M, and DMEM in the presence of M-CSF for 48 h. The metabolic activity of the cells was analyzed by the MTT assay and is presented relative to the activity of cells treated with DMEM alone (*n* = 3). **(B,C)** Flow cytometric analysis of annexin V/propidium iodide (PI) staining of BMDMs treated with ESC-M, Con-M, DMEM with M-CSF, and DMEM alone for 24 h, respectively (*n* = 3). **(D)** Effect of ESCs on caspase-9 activation. BMDMs were treated with Con-M and ESC-M for 12 and 24 h, respectively and whole cell lysates were analyzed by Western blotting for caspase-9 activation. **(E)** IL-34 in BMDMs and ESCs was detected by Western blot analysis. *p < 0.05, two-sided Wilcoxon test. Data are represented as mean ± SEM.

The ESC-induced macrophage distribution could result from either increased recruitment of these cells into the teratoma or cell survival. We first examined the function of ESCs in macrophage growth. It has been well-documented that M-CSF is a hematopoietic growth factor necessary for monocyte survival, proliferation, and differentiation (32, 33). L929 conditioned medium is the source for M-CSF. Mouse ESC-CM (without direct cell–cell contact) and control medium (Con-M, medium to culture ESCs) were used in the study. Day 7 BMDMs were cultured with DMEM alone, Con-M and ESC-M and DMEM with M-CSF for 48 h and cell viability was measured by monitoring metabolic activity of the cells using MTT assay. Incubation of BMDMs in the presence of M-CSF and ESC-M caused a significant increase in cell viability compared to medium without M-CSF (DMEM alone) and Con-M treatment (Figure 2A). These data suggest that ESC-M can partially prevent the loss of macrophage viability after M-CSF withdrawal, although to a lesser extent than did M-CSF. Furthermore, heat-inactivated ESC-M by boiling for 10 min failed to increase macrophage viability (data not shown).

### ESCs protect macrophages from M-CSF withdrawal-induced apoptosis *in vitro*

To further evaluate whether the survival effect of ESCs was due to the inhibition of apoptosis, apoptosis of macrophages was assessed by surface Annexin V staining using FACS and caspase-9 activation. When mature BMDMs were cultured with M-CSF, <10% of cells were Annexin V-positive (Figures 2B,C). However, when cells were subjected to medium without M-CSF or Con-M, more than 25% of the cells underwent apoptosis (Figures 2B,C). Propidium iodide (PI) staining revealed that M-CSF withdrawal or Con-M treatment for 24 h did not increase PI-positive macrophages significantly compared to M-CSF or ESC-M treatment (Figure 2C). Furthermore, Con-M treatment induced activation of caspase-9, assessed by the appearance of the cleaved caspase-9 (Figure 2D). Treatment of cells with ESC-M significantly protected M-CSF withdrawal-induced caspase-9 activation and apoptosis (Figures 2B–D). These results suggest that the anti-apoptotic effect of ESCs on macrophages resulted in enhanced cell survival. In order to identify whether ESC-mediated cell survival is induced directly by mediators in ESC-M or indirectly by stimulating the release of secondary mediators acting in an autocrine manner, we detected M-CSF and IL-34, the two most well-documented cytokines that regulate macrophage survival and differentiation (34). ESCs did not produce M-CSF (data not shown) but expressed a high level of IL-34 (Figure 2E) and secreted into the ESC-M (16.034 ± 4.56 ng/ml, *n* = 3). BMDM treated with ESCs did not further increase M-CSF and IL-34 expression (data not shown), suggesting that ESC-induced BMDM survival is M-CSF-independent and that IL-34 from ESCs may promote macrophage survival.

### Activation of PI3K/Akt and ERK is necessary for ESC-induced macrophage survival

**Figure 3:**
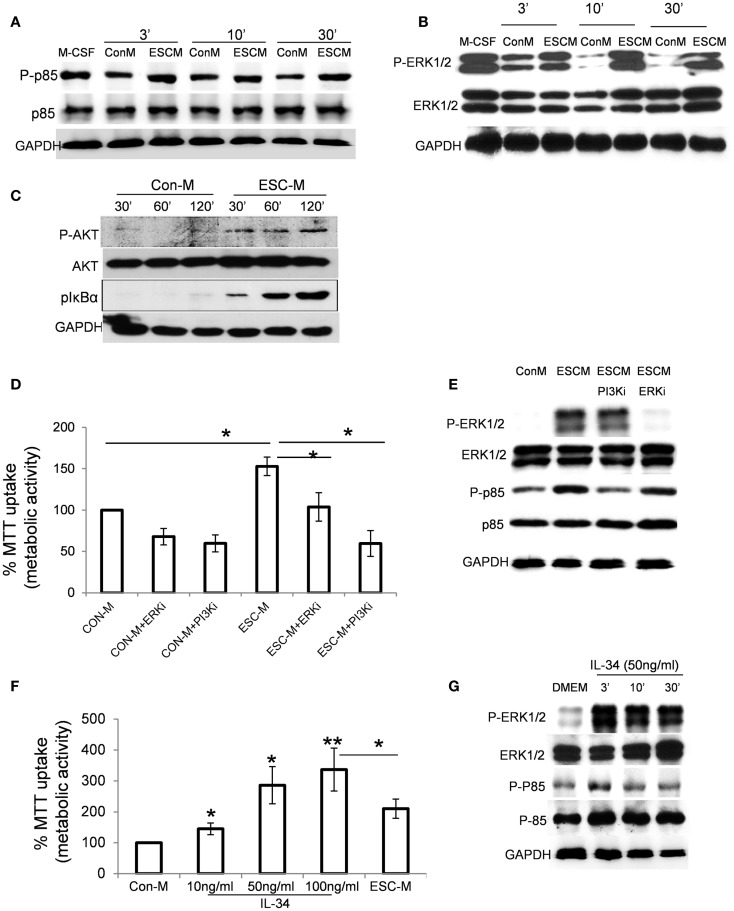
**Effect of ESCs on activation of PI3K/Akt, ERK1/2, and NF-κB pathways**. Phosphorylation of p85 **(A)**, p-ERK1/2 **(B)**, and pAkt and NF-κB **(C)** in BMDMs treated with Con-M and ESC-M for the indicated time. **(D)** Effect of inactivation of PI3K and ERK1/2 in ESC-induced cell survival. BMDMs were pretreated with PI3K inhibitor LY-294002 (20 μM) and ERK inhibitor PD98059 (10 μM) for 30 min and then treated with Con-M and ESC-M for 48 h. The metabolic activity of the cells was analyzed by the MTT assay and is presented relative to the activity of cells treated with Con-M (*n* = 3, *p < 0.05, two-sided Wilcoxon test. Data are represented as mean ± SEM). **(E)** BMDMs were pretreated with PI3K inhibitor LY-294002 (20 μM) and ERK inhibitor PD98059 (10 μM) for 30 min and then treated with Con-M and ESC-M for 30 min. Phosphorylation of p85 and p-ERK1/2 was detected by Western Blot assay. **(F)** Effect of IL-34 on macrophage survival. BMDMs were incubated with mouse recombinant IL-34 at the indicated concentration for 48 h. The metabolic activity of the cells was analyzed by the MTT assay and is presented relative to the activity of cells treated with Con-M (*n* = 3, *p < 0.05; **p < 0.001, ANOVA, data are represented as mean ± SEM). **(G)** Phosphorylation of p85 and p-ERK1/2 in BMDMs treated with IL-34 (50 ng/ml) for the indicated time.

The PI3K pathway is one of the most potent intracellular mechanisms for promoting cell survival, and PI3K/Akt and extracellular regulated kinase1/2 (ERK1/2) regulate macrophage survival in response to M-CSF and IL-34 (35–37). We therefore examined whether activation of PI3K and ERK1/2 was required for ESC-mediated macrophage survival. As shown in Figures 3A,B, ESC-M stimulated phosphorylation of p85 regulatory subunit of PI3K and ERK1/2 as early as 3 min, and a persistent phosphorylation level was maintained up to 30 min. ESCs also induced activation of the major downstream kinase Akt of PI3K by phosphorylating residue Ser473 (Figure 3C). It is interesting to note that ESC-M also induced NF-κB activation by enhancing IκBα phosphorylation (Figure 3C). We further determined whether LY-294002 and PD98059, specific inhibitors of PI3K and ERK1/2, can reverse the protective role of ESC-M on macrophage survival. Both LY-294002 and PD98059 significantly reduced ESC-mediated macrophage survival (Figure 3D), suggesting that PI3K and ERK1/2 activation are required for ESC-mediated macrophage survival. Moreover, the activation of PI3K and ERK1/2 was inhibited by LY-294002 and PD98059, respectively, which parallels their effect on macrophage survival (Figure 3E).

We showed that ESCs produce IL-34 (Figure 2E) and the concentration of IL-34 in ESC-M was 16.034 ± 4.56 ng/ml. We therefore examined the ability of mouse recombinant IL-34 to promote macrophage survival on BMDMs. IL-34 simulated macrophage survival in a dose-dependent manner (Figure 3F). IL-34 at low as 10 ng/ml increased macrophage survival. Furthermore, IL-34 stimulated phosphorylation of ERK1/2 and PI3K in macrophages to a similar degree, with similar kinetics, compared to ESC-M treatment (Figure 3G).

### ESCs induces typical shape change

**Figure 4:**
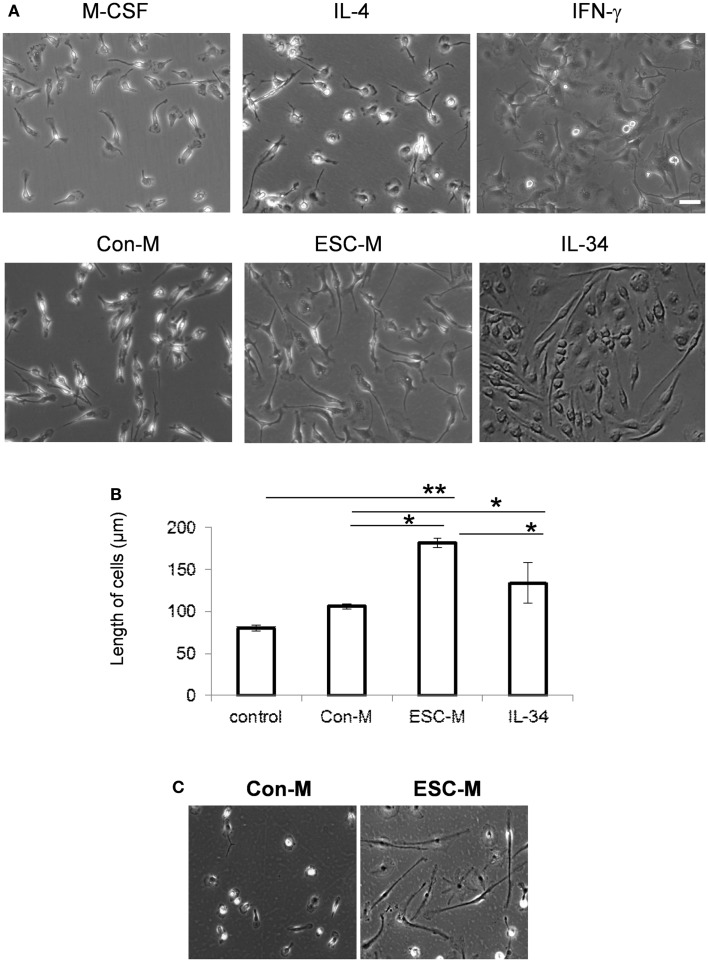
**Effect of ESCs on macrophage activation**. **(A)** Representative phase-contrast photomicrographs of BMDMs (original magnification, ×400). BMDMs were cultured with M-CSF, IL-4, IFN-γ, IL-34, Con-M, and ESC-M for 72 h. **(B)** Length of BMDMs incubated with IL-34, Con-M, and ESC-M for 72 h was measured by ImageJ (*n* = 6, *p < 0.05, **p < 0.001, two-sided Wilcoxon test. Data are represented as mean ± SEM). **(C)** Representative phase-contrast photomicrographs of primary microglial cells incubated with Con-M and ESC-M for 72 h. Original magnification, ×400.

We next examined how ESCs modulate macrophage function. We exposed mature BMDMs to interferon γ (IFN-γ), IL-4, ESC-M, Con-M, and IL-34 for 72 h, respectively. BMDMs present a unique morphology, depending on the stimulation used. Cells treated with IL-4, which stimulates M2 activation, adopted a spindle-shape morphology (Figure 4A). M1 macrophages induced by IFN-γ had a relatively round shape with large filopodia. Con-M treatment exhibited the typical bipolar, spindle-shaped morphology of BMDMs. In contrast, ESC-M led to a majority of elongated fibroblast-like-shaped cells and some of the macrophages showed a long, single process or bipolar processes (Figure 4A). IL-34 treated cells demonstrated a wider range of cell length whereas the ESC-M treated cells displayed a relatively shorter range because the cells were all approximately the same length (Figure 4A). Quantitative analysis showed that ESC-M-treated macrophages exhibited a significantly higher degree of elongation compared either to Con-M treated or untreated macrophages (Figure 4B). The average length of cells treated with IL-34 was longer than that of Con-M treated cells but shorter than that of ESC-M, suggesting that other factors produced by ESC-M may have contributed to cell elongation. To further demonstrate that the ESC-induced phenotypic characteristic was not restricted to BMDMs, we isolated primary microglial cells from brain. ESC-M also resulted in remarkable elongation in microglial cells (Figure 4C).

### ESCs maintain macrophage phenotype and function

**Figure 5:**
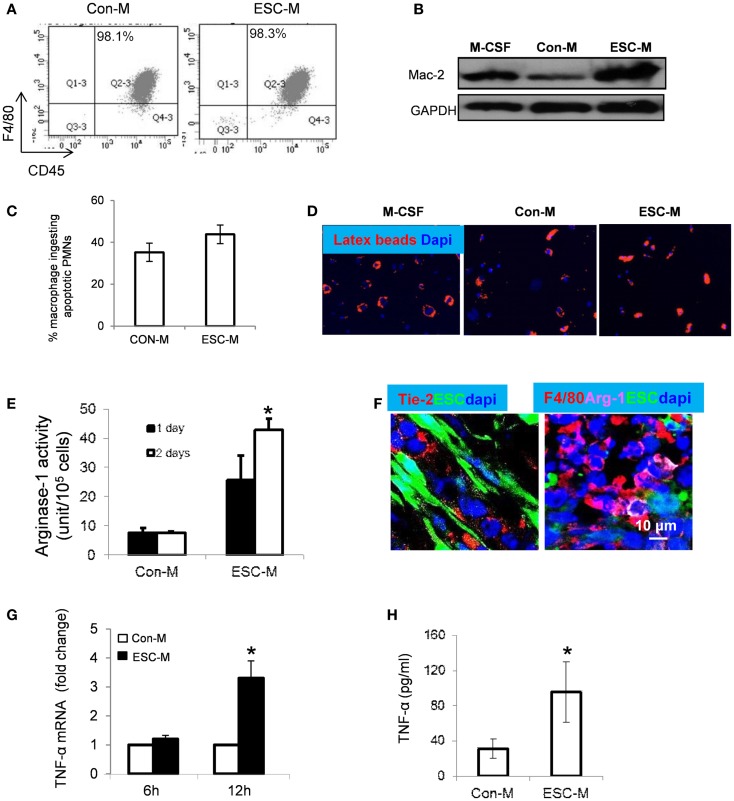
**Effect of ESCs on macrophage activation**. BMDMs were incubated with Con-M or ESC-M for 72 h and F4/80 (macrophage marker), CD45 (hematopoietic marker), and Mac-2 (macrophage marker) were assessed by flow cytometry **(A)** and Western Blot **(B)**, respectively. **(C)** BMDMs were pretreated with Con-M and ESC-M for 48 h and then incubated with apoptotic neutrophils for 30 min. The number of macrophages ingesting apoptotic cells was counted (*n* = 4, data are represented as mean ± SEM). **(D)** BMDMs pretreated with Con-M and ESC-M ingestion of latex beads (red, original magnification, ×400). **(E)** BMDMs were treated with Con-M and ESC-M for 1 and 2 days and arginase-1 activity was detected by colorimetric assay (*n* = 3, *p < 0.05, two-sided Wilcoxon test. Data are represented as mean ± SEM). **(F)** Representative confocal images of immunostaining of sections from mice at 2 weeks after ESC injection showing positive staining for Tie-2 (red, left) and F4/80 (red, right) Arginase-1 (purple), respectively. **(G)** TNF-α mRNA in BMDMs treated with Con-M and ESC-M for 6 and 12 h was detected by real time RT-PCR (*n* = 4, *p < 0.05, two-sided Wilcoxon test. Data are represented as mean ± SEM). **(H)** TNF-α in the supernatants of BMDMs treated with Con-M and ESC-M for 48 h was detected by ELISA (*n* = 3, *p < 0.05, two-sided Wilcoxon test. Data are represented as mean ± SEM).

In order to know whether ESC treatment would maintain macrophage phenotype and function, we analyzed the expression of macrophage markers such as F4/80 and Mac-2 on cells treated with ESC-M. BMDMs were treated with Con-M and ESC-M for 3 days and expression of macrophage markers was confirmed by flow cytometry and Western Blot analysis, respectively. Both Con-M and ESC-M treatment maintained macrophages expressing a high level of F4/80 (Figure 5A). Mac-2 expression was enhanced by ESC-M treatment and the expression level was higher compared with Con-M treatment (Figure 5B). To further study, whether ESC-M-treated macrophages were biologically functional, we incubated BMDMs with apoptotic neutrophils (PMNs) and latex beads for 30 min. The results showed that ESC-M-treated BMDMs displayed active functional phagocytosis of apoptotic cells and latex beads (Figures 5C,D).

### ESCs polarize macrophages into unique M2-like cells

In addition to the role of ESCs promoting macrophage infiltration and survival, ESCs are able to activate BMDMs and stew them toward the M2 phenotype (Arginase-1^high^YM1^high^) via activation of STAT3 and STAT6 pathways (24). A recent study showed that macrophage elongation enhanced the effect of M2-inducing cytokines and inhibited the effect of M1-inducing cytokines, suggesting that cell shape has an important role in modulating macrophage activation (38). We demonstrated in the present study that the phenotypic characterizations of ESC-treated macrophages were distinct from classic M2 macrophages induced by IL-4 (Figure 4A). We thus further evaluated whether ESC-treated macrophages are different from “alternatively activated” M2 macrophages. Treatment with ESC-M significantly enhanced arginase-1 (Arg-1) activity in BMDMs in a time-dependent manner, compared to treatment with Con-M using a colorimetric assay that detects production of urea (Figure 5E). The distributions of angiopoietin (Ang) receptor (Tie)-2^+^ (Tie-2) cells and F4/80^+^/arginase-1^+^ macrophages were detected in the teratoma *in vivo* (Figure 5F). It has been shown that M2 express a very low level of TNF-α (39, 40). However, we showed that macrophages expressed only minimal TNF-α mRNA in the absence of ESC-M (Figure 5G). Upon co-culture with ESC-M, TNF-α expression increased significantly in macrophages (Figure 5G). Furthermore, the amount of TNF-α secreted into the culture medium was significantly increased in BMDMs treated with ESC-M compared to the amount present in supernatants of Con-M-treated macrophages (Figure 5H). In summary, ESC-macrophages exhibited an Arg-1^high^Tie-2^high^TNF-α^high^ phenotype, which differs from conventional M2 phenotypes.

### ESCs exerts angiogenic activity *ex vivo* and *in vivo*

**Figure 6:**
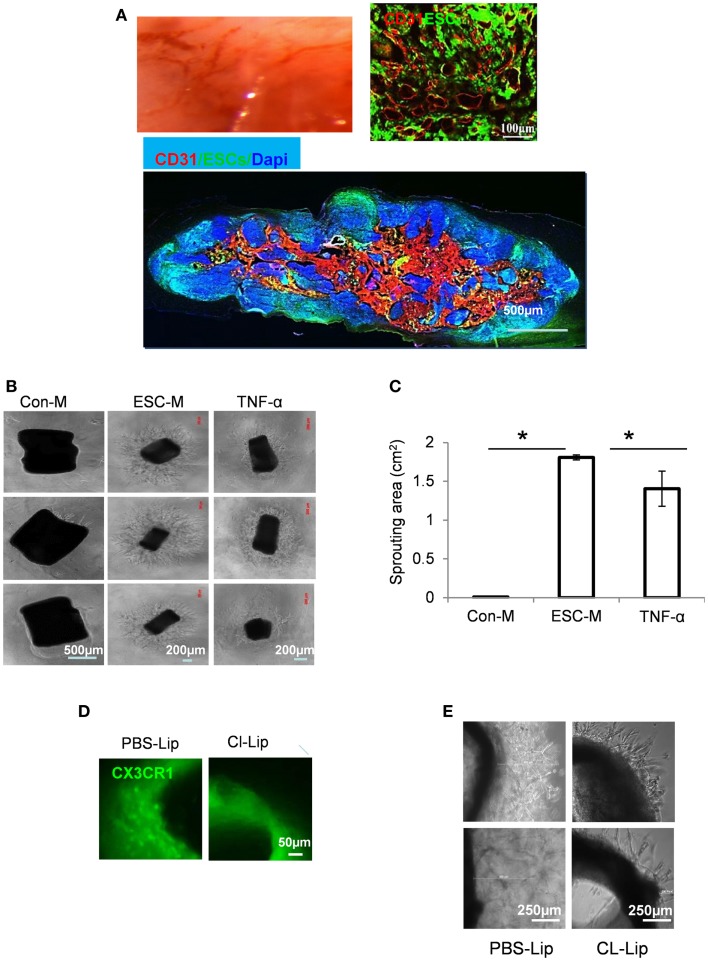
**Embryonic stem cells induced an angiogenic switch in macrophages**. **(A)** Representative gross morphology micrograph showing blood vessel development during teratoma progression (top left) and immunostaining by endothelial marker CD31 (red) in sections from mice at 3 weeks after ESC injection (top right and bottom). **(B)** Representative gross morphology of aortic rings in Matrigel containing Con-M (left panel), ESC-M (middle panel), or TNF-α at 10 ng/ml (right panel) at day 6. **(C)** Area of vascular sprouting at day 6 in Con-M, ESC-M, and TNF-α was measured by ImageJ (*n* = 6, *p < 0.05, two-sided Wilcoxon test. Data are represented as mean ± SEM). **(D)** Representative gross morphology of aortic ring from CX3CR1^GFP/+^ mice treated with PBS-Lip (left) and CL-Lip for 4 weeks (right). **(E)** Mice were treated with PBS-Lip (left) and CL-Lip (right) for 4 weeks and aortic rings were cultured in Matrigel containing ESC-M for 6 days. Representative gross morphology of aortic ring sprouting was taken.

It is widely accepted that tumor growth requires angiogenesis. Therefore, fast teratoma growth is supposedly induced by increased angiogenesis. ESCs were injected into the spinal cord and images were taken at 3 weeks after cell transplantation. Spinal cords with teratoma appeared reddish or brownish, suggesting an increased permeability (Figure 6A). Immunohistochemical analysis of teratoma tissue with anti-CD31 antibody showed a massively branched intratumoral vascular network at 3 weeks after cell injection (Figure 6A). This high density of “plexus-like” vascularity in teratoma may be important for teratoma growth. By contrast, injection of PBS alone in the spinal cord did not produce neovascularization (data not shown).

To better understand the contribution of macrophages to vascular development during teratoma progression, we performed a ring sprouting *ex vivo* assay. A 3D-culture of aortic rings in Matrigel was used to evaluate the outgrowth of linear endothelial structures from the preexisting vessel (41). The aorta ring assay is thought to more closely mimic multiple stages of *in vivo* angiogenesis, including endothelial cell proliferation, migration, and tube formation. Mouse thoracic aorta was sectioned into 1-mm rings, and incubated in growth factor-reduced matrigel with Con-M or ESC-M for 6 days. Sprouting from the rings was photographed and outgrowth area was quantitated. ESC-M treatment significantly increased the areas of sprouting (1.81 ± 0.03 mm^2^) at 6 days, whereas Con-M-treated ring segments showed little sprouting (Figures 6B,C). Qualitatively, the arborization of endothelial networks emanating from aortic rings was also more complex in the rings treated with ESC-M. Together, these data demonstrated an important role for ESC-mediated angiogenesis in aortic rings *ex vivo*.

We showed that ESC-educated macrophages (SEM) exhibited an Arg-1^high^Tie-2^high^TNF-α^high^ phenotype (Figures 5E–H). In order to know whether TNF-α is responsible for ESC-enhanced angiogenesis, aortic rings were cultured with TNF-α at 10 ng/ml for 6 days. A significant increase in angiogenic spouting was observed in aortic rings in response to TNF-α treatment (Figures 6B,C). Therefore, we consider that TNF-α contributed, at least partially, to ESC-induced angiogenesis.

It has been shown that macrophages are found around sprouting neovessels and are particularly abundant at the root of the vascular outgrowth (42). Pharmacologic ablation of macrophages from aortic explants blocked formation of neovessels *in vitro* and reduced aortic ring-induced angiogenesis *in vivo* (42). We further determined how crucial macrophages are to the enhanced angiogenic ability of ESCs. We applied a well-documented approach to deleted macrophages by treating mice with liposome-encapsulated clodronate (Cl-Lip) or control liposomes (PBS-Lip) (43). We used CX3CR1 ^GFP/−^ mice, in which one (CX3CR1^GFP/+^) copy of the CX3CR1 gene was interrupted by EGFP (44). CX3CR1 is highly expressed by human and mouse macrophages (45). Intraperitoneal injection of mice with Cl-Lip but not control liposomes (PBS-Lip) resulted in complete depletion of macrophages in the aortic ring tissue (Figure 6D). Depletion of macrophages by Cl-Lip led to a markedly reduced angiogenic response to ESC-M (Figure 6E). However, we cannot rule out the direct effect of ESC-M on aortic ring sprouting, because Cl-Lip treatment did not completely inhibit the vascularization (Figure 6E).

### Targeting macrophages inhibits ESC-induced angiogenesis and teratoma development

**Figure 7:**
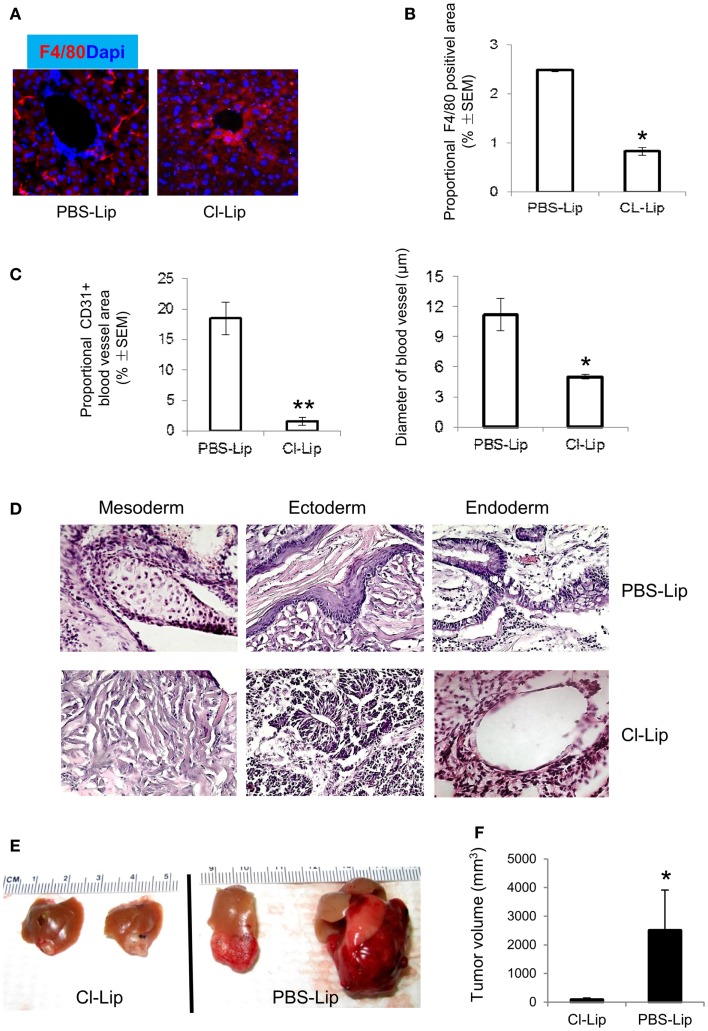
**Depletion of macrophages inhibiting angiogenesis and teratoma growth**. **(A)** Cl-Lip was administrated i.p. at the day for ESC injection and twice a week for 4 weeks. PBS-Lip was used as control. Liver tissues were stained with F4/80. Note that Cl-Lip treatment completely depleted F4/80^+^ macrophages in liver harvested from the Cl-Lip-treated group (left) and control group (treated with PBS-Lip) at 4 weeks after ESC transplantation. Quantification of F4/80^+^ macrophages **(B)**, density of blood vessels stained for CD31 [**(C)**, left], size of blood vessels [**(C)**, right], HE staining **(D)**, and representative images of teratomas **(E)** in teratomas from Cl-Lip- or PBS-Lip-treated mice at 4 weeks after ESC transplantation. **(F)** Average teratoma size at 4 weeks after ESC transplantation in the Cl-Lip and PBS-Lip treated groups. *p < 0.05, **p < 0.001, *n* = 5, two-sided Wilcoxon test. Data are represented as mean ± SEM.

A large amount of macrophage infiltration and phenotype of M2-like macrophages in the teratoma suggested that macrophages may create a microenvironment for teratoma development. We depleted macrophage populations from mice to verify the contribution of macrophages to teratoma growth. We demonstrated that i.p. injection of Cl-Lip twice a week after ESC transplantation into liver resulted in near-complete depletion of macrophages in liver and teratoma when assayed at 4 weeks (Figures 7A,B). We also quantified the blood vessel density by counting the percentage of the area occupied by cross-section of all blood vessels in an image. We observed that blood vessel density in teratoma from mice treated with Cl-Lip was significantly lower than that of control treatment (Figure 7C). Blood vessels were significantly smaller in Cl-Lip-treated teratoma compared to control treatment (Figure 7C). Depletion of macrophages did not affect the pluripotency of ESCs, as all three germ layers can be observed in macrophage-deleted teratoma (Figure 7D). Teratomas from mice treated with PBS-Lip appeared much darker and were filled with blood, indicating that functional vasculature had formed via angiogenesis (Figure 7E). In contrast, tumor tissue from mice treated with Cl-Lip was transparent (Figure 7E). Subsequently, ablation of macrophages significantly inhibited teratoma growth, with a mean tumor size of 83.13 ± 60.81 mm^3^ in Cl-Lip group vs. 2502.75 ± 1410.02 mm^3^ in mice treated with PBS-Lip (*n* = 5, *p* < 0.05, Figure 7F).

## Discussion

In this study, we demonstrated for the first time that BMDMs can be guided by ESCs to migrate to the site of ESC implantation while soluble factor(s) produced by ESCs polarize macrophages into a novel Arg-1^high^Tie-2^high^TNF-α^high^ phenotype. Furthermore, ESCs can prevent BMDM apoptosis induced by M-CSF-withdrawal through PI3K/Akt and ERK1/2 activation. We demonstrated that these ESC-educated macrophages (SEM) exhibit an elongated morphology and produce high level of TNF-α, which participates in angiogenesis and contributes to teratoma progression. Depletion of macrophages completely inhibits ESC-induced angiogenesis and teratoma development. These studies provide a novel rationale for the control of teratoma development by targeting macrophage growth or activation.

Macrophage colony-stimulating factor is known to regulate monocyte/macrophage survival (32, 33). PI3K/Akt and ERK or MARK are the major signaling pathways triggered by M-CSF stimulation (35, 46, 47). We showed that ESCs promote the survival of cultured BMDMs after M-CSF withdrawal, and that activation of both PI3K/Akt and ERK1/2 MAP kinase are required for the survival effect of ESCs, which is similar to that activated by M-CSF. ESC-maintained macrophage survival and function was independent of M-CSF, because M-CSF was detectable neither in ESC-M nor in ESC-M-treated macrophages (24). In the present study, we studied whether other factors are involved as autocrine or paracrine effectors to induce macrophage survival. For example, GM-CSF and IL-4 are known to regulate monocyte/macrophage survival (48). Our results ruled out the requirement for GM-CSF and IL-4, as these cytokines were undetectable in ESC-M (24). Recent studies reported that IL-34 is an alternative ligand for M-CSF receptor (CSF-1R) (34, 49). IL-34 binds specifically to human and mouse myeloid cells, induces ERK1/2 activation, and supports macrophage proliferation and differentiation (34). We showed that IL-34 is highly expressed by ESCs. IL-34 promotes macrophage survival and activates ERK and PI3K pathways. Although IL-34 also results in cell elongation, its effect is not as strong as that of ESC-M. It is possible that other soluble factors produced by ESCs play a role in cell shape change. It has been reported recently that IL-34-activated macrophages exhibit an IL-10^high^ IL-12^low^ M2 profile in response to LPS stimulation (50). Therefore, it is likely that IL-34 produced by ESCs play a pivotal role in ESC-induced macrophage survival and M2 polarization. More studies are needed to investigate whether IL-34 secretion and M2-like polarization of macrophages are general features of ESCs.

It is interesting to note that a large number of macrophages exist in the early stage of teratoma development and the number of macrophages rapidly declines at 2 weeks after ESC injection. We reasoned that ESCs have ability to regulate macrophage survival and activation. The inhibition of macrophage apoptosis by ESCs at an early stage may favor teratoma initiation and development. However, the effect of ESCs on macrophage may be reduced once ESCs are differentiated into its three germ layer structures. It will be important to determine whether fully differentiated tissues lose the ability to maintain macrophage function or have the capacity to inhibit macrophage survival. Beside IL-34, the factors produced by ESCs that promote macrophage growth and program M2-like phenotype remain unknown. It is possible that the function/phenotypes of macrophages are regulated by coordinated action of different classes of molecules secreted by ESCs. However, more detailed studies will be necessary to determine whether additional molecules either alone or in combination contribute to macrophage survival. The identification of these factors appears crucial in the development of strategies to prevent and/or reverse macrophage phenotype and thereby increase the safety of stem cell applications in clinical settings.

Beside mediation of macrophage survival, ESCs are able to regulate macrophage activation. It is well-documented that M1 macrophages express high levels of nitric oxide (NO), reactive oxygen species (ROS), and TNF-α, contributing to tissue inflammation and damage. In contrast, M2 macrophages produce anti-inflammatory factors and have a reduced capacity to produce pro-inflammatory molecules, thereby contributing to wound healing and tissue remodeling, as well as tumor progression (51–53). Tumor microenvironment educates macrophages to perform supportive roles that initiate and promote tumor progression (28). Tumor-associated macrophages (TAMs) have many properties of M2 phenotype such as impaired expression of IL-12 and TNF-α, and up-regulated levels of M2 markers including Arg-1 and YM1 (54–58). However, TAMs from several tumor models also exhibit typical M1 cytokines such as TNF-α and IL-1β (59). We previously showed that ESC-treated macrophages express higher levels of M2 markers such as PPAR-γ, Arg-1, YM1, as well as M2 cytokines including VEGF, MMP9, and MCP-1 (24), suggesting that ESCs induced the M2-like phenotype. In the present study, we demonstrated that ESCs not only increased Arg-1 and Tie-2^+^ expression but also triggered TNF-α expression, implying that ESC-educated macrophages are different from classic M2 macrophages and resemble more TAMs. ESCs–macrophages not only exhibited M2 characteristics (expression of M2 markers and STAT3/6 activation) but also acquired properties of M1 macrophages (activation of NF-κB) and TAMs (high levels of Tie-2 and TNF-α), exhibiting enhanced neovasculation in an *in vitro* and *ex vivo* angiogenesis assay. Another point of interest is that ESCs also activate the NF-κB pathway. Defective NF-κB activation within macrophages leads to the development of an M2 activation. Although ESCs are not oncogenically transformed, they have potent ability to regulate macrophage function and induce the unique Arg-1^high^Tie-2^high^TNF-α^high^ phenotype. Tie-2-expressing monocytes/macrophages (TEM) share some characteristics with M2 macrophages and are highly pro-angiogenic cells critical for tumor vascularization (59, 60). Tie-2 expression can be up-regulated by TNF-α (61). Specific depletion of TEM or conditional *Tie2* knockdown inhibits tumor angiogenesis. (62–64). Within the ESC implantation site, the presence of Tie-2^+^ macrophages and TNF-α secreted by ESC-macrophages stimulates angiogenesis and supports teratoma growth. Several angiogenic molecules may be linked to ESC-induced angiogenesis. We previously showed that ESCs increased macrophage MIF, MMP9, VEGF, and MCP-1. MIF secretion is tightly regulated by TNF-α (65, 66) and MIF can also increase TNF-α expression (67, 68). Therefore, TNF-α may be the key factor in ESC-induced angiogenesis. Anti-TNF-α agents such as infliximab (IFX), etanercept (ETA), adalimumab (ADA), golimumab (GLM), and certolizumab pegol (CZP) have been widely used for the treatment of a variety of chronic inflammatory diseases. Thus targeting TNF-α by administration of TNF-α antagonists may be a promising option to suppress teratoma angiogenesis. However, side effects such as increasing frequency of infection and promoting tumor growth by induction of T cell apoptosis make anti-TNF-α treatment a difficult balance. Administration of CZP can minimize this side effect since it does not induce T cell apoptosis but remains an efficacious treatment for inflammatory diseases because of the lack of an Fc region (69).

Embryonic stem cells can attract macrophages, induce M2 activation, and promote macrophage survival, and consequently, inhibition of any of these three functions could potentially offer a therapeutic solution to prevent teratoma development. A few strategies are developed to target macrophages: inhibiting macrophage migration, suppressing macrophage survival, promoting M1 activation, and blocking M2 polarization (70). Our data suggested that IL-34 maybe important to maintain macrophage survival via activation of PI3K and ERK1/2. Therefore, targeting IL-34 and the PI3K/ERK pathways could decrease macrophage number effectively and alter the microenvironment involved in teratoma angiogenesis and development. Because IL-34 was recently discovered (34), no antagonists are currently available to inhibit IL-34 activity. Thus, in turn, the antagonists of IL-34 receptor (CSF-1R) can be applied to block IL-34 binding to its receptor. Anti-CSF-1R treatment to inhibit tumor growth *in vitro* and *in vivo* has been well-documented (71, 72). Similar strategies can be applied to target macrophages in teratoma models. In addition, combined targeting of the ERK1/2 and PI3K pathways in teratoma may be a potential therapeutic strategy. Numerous small molecule inhibitors of specific PI3K and ERK1/2 pathways have been developed to exhibit promising anti-tumor activity *in vitro* and *in vivo* (73). For example, therapy with a dual PI3K (ZSTK474) and MEK inhibitor (CI-1040) combination is more effective than either inhibitor alone in cancer treatment (74). Combination of the PI3K inhibitor GDC-094 and the MEK inhibitor PD 0325901 induced marked tumor growth inhibition *in vivo* (75). Further study is required to demonstrate whether the dual PI3K and ERK inhibition have anti-teratoma activity *in vivo*, either through direct inhibition of macrophage survival, or ESC growth because PI3K is implicated in regulation of ESC proliferation (76).

Specifically, targeting M2 or TAM-like cells remains challenging. It has been shown that pharmacological skewing of TAM polarization from an M2 macrophage phenotype to a full M1 macrophage phenotype sustains anti-tumor immunity (57). It is possible to re-polarize TAMs. The recent report showed that M2pep, a peptide, can preferentially binds to M2 macrophages with low affinity for other leukocytes. Systemical administration of an M2pep fusion peptide with a proapoptotic peptide specifically reduced M2-like macrophages (77). A combination of CpG oligodeoxynucleotides and an IL-10 receptor-specific antibody switched TAMs from an M2 to an M1 type and triggered an innate response that was able to cure the majority of mice bearing large tumors (78).

One therapeutic option is to target STAT3. Numerous studies demonstrated that constitutive activation of STAT3 promotes initiation and development of tumors by inducing cell proliferation, angiogenesis, and metastasis in a wide variety of tumors (79). Furthermore, STAT3 is a critical mediator of LIF-induced signaling pathways that regulate ES cell self-renewal and proliferation (80). STAT3 also contributes to M2 macrophage activation. Therefore, STAT3 could be an attractive target to control teratoma development by direct effects on ESC growth and macrophage M2-like activation. Numerous strategies to suppress STAT3 activation have been developed such as anti-sense oligonucleotide targeting STAT3, synthetic drugs, small molecules, and gene therapy techniques (79).

In conclusion, our present findings show an important link between ESC-induced macrophage infiltration, growth and activation, initiation of angiogenesis, and teratoma development. ESCs induce BMDM accumulation and stew novel pro-angiogenic phenotype and thus accelerate teratoma development. A better understanding of the regulation and function of macrophages in the tumorigenicity of ESCs may yield useful therapies for the safe transplantation of ESCs. Targeting of the host microenvironment of the transplantation site such as modulating macrophage phenotype and function rather than ESCs directly could be a more efficient approach for suppressing angiogenesis and teratoma progression without affecting the pluripotency of ESCs.

## Author Contributions

The author(s) have made the following declarations about their contributions: conceived and designed the experiments: Yi Ren. Performed the experiments: Tianxiang Chen, Xi Wang, Lei Guo, Mingmei Wu, Zhaoxia Duan, Jing Lv, Wenjiao Tai, Hemamalini Renganathan, and Ruth Didier. Analyzed the data: Tianxiang Chen, Xi Wang, Jianqing Fan, and Yi Ren. Contributed reagents/materials/analysis tools: Jinhua Li, Xiaoming Chen, Dongming Sun, Xijing He, Jianqing Fan, and Wise Young. Wrote the first draft of the paper: Yi Ren. Contributed to the writing of the paper: Yi Ren.

## Conflict of Interest Statement

The authors declare that the research was conducted in the absence of any commercial or financial relationships that could be construed as a potential conflict of interest.
